# Long-Term Outcomes of Concomitant Modified Cox-Maze and Mitral Surgery

**DOI:** 10.5761/atcs.oa.24-00119

**Published:** 2025-03-20

**Authors:** Choosak Kasemsarn, Pramote Porapakkham, Sahaporn Wathanawanichakun, Piyawat Lerdsomboon, Krisulang Chanpa

**Affiliations:** Department of Cardiothoracic Surgery, Central Chest Institute of Thailand, Nonthaburi, Thailand

**Keywords:** atrial fibrillation, surgical biatrial RF ablation, modified Maze procedure, long-term outcome

## Abstract

**Purpose:** There are limited data on outcomes of combined Maze and mitral valve procedures beyond 10 years. This study analyzed the efficacy of this operation.

**Methods:** Between June 2004 and December 2022, 406 patients underwent mitral surgery concomitant with Maze procedure were evaluated. Rhythm outcomes, predictors of recurrence, and survival were assessed.

**Results:** The median follow-up period was 100 months. Rheumatic disease was present in 58%. Mitral valve repair was performed in 57.1%. Freedom from atrial fibrillation (AF) at 5, 10, and 15 years was 82.5%, 70.8%, and 52.7%, respectively. Overall survival rates were not different between patients in sinus rhythm (SR) and those who remained in AF (p = 0.172). However, patients in SR experienced fewer neurological complication (p = 0.001). Predictors of AF recurrence included preoperative AF duration (p = 0.005), left atrial diameter (LAD) >50 mm (p <0.001), concomitant tricuspid valve surgery (p = 0.049), and the presence of AF on postoperative day 7 (p <0.001). Factors influencing survival were age >60 years (p <0.001) and a postoperative left ventricular ejection fraction <40% (p <0.001).

**Conclusions:** The combined Maze and mitral valve surgery provides significant benefits in managing AF with mitral disease. Predictors of recurrence included AF duration, LAD size >50 mm, associated tricuspid valve disease, and AF on day 7. SR patients had fewer neurological complications.

## Introduction

Atrial fibrillation (AF) is the most common arrhythmia in humans, with 70% of cases associated with organic heart conditions.^1)^ This electrical disturbance significantly impacts quality of life, particularly due to an increased risk of stroke, and adversely affects both early and long-term outcomes.^2)^ In 40%–60% of AF patients, the abnormality results from mitral valve pathologies,^3)^ a prevalence significantly higher than that associated with other heart valve diseases.^1)^ Clinical outcomes are often poorer if only the mitral defect is corrected while AF persists.^4,5)^

The classical Cox-Maze procedure is considered the gold-standard operation to eliminate this abnormal electrical disturbance. Modern advancements have led to the development of a modified Maze procedure using alternative energy sources. These devices achieve success rates comparable to the classical “cut-and-sew” technique in restoring normal sinus rhythm (NSR).^3)^ However, long-term freedom from AF after this procedure varies depending on the type of AF, with concomitant mitral valve surgery yielding the worst outcomes at 10 years (64%, 62%, and 41% for paroxysmal AF, chronic AF, and concomitant cases, respectively).^6)^ The etiology of mitral valve disease concomitant with AF is another interesting issue. One paper reported rheumatic disease is one of the predictors for failure of the Cox-Maze procedure,^7)^ while others paper^8–11)^ demonstrated no significant differences in outcome between rheumatic and nonrheumatic mitral valve.

Long-term data on the outcomes of concomitant Cox-Maze and mitral valve surgery beyond 10 years remain limited. A study with a 10-year follow-up reported freedom from AF in 82% of stand-alone Cox-Maze and 75% of concomitant Cox-Maze (p = 0.77), a majority of this combination were mitral valve surgery (71%).^12)^ Another study demonstrated AF cure rates of 63% and 51% at 10 and 20 years, respectively.^13)^ This study aims to investigate outcomes in a sizable cohort undergoing the combined procedure, with a focus on long-term efficacy and predictors of success.

## Patients and Methods

The study included 406 patients who underwent concomitant mitral valve surgery with a modified biatrial Maze operation at the Central Chest Institute of Thailand between June 2004 and December 2022. Inclusion criteria for the combined procedures required patients to have mitral valve disease associated with permanent AF. Exclusion criteria included New York Heart Association (NYHA) class IV, redo cases, left ventricular ejection fraction (LVEF) <30%, left atrial diameter (LAD) >70 mm (measured as M-mode anteroposterior dimension by transthoracic echocardiography), or severe left atrial wall calcification. All patients exhibited AF on electrocardiogram (EKG) for more than 6 months prior to surgery, with no reversibility to SR, defining their condition as permanent AF per guidelines by the American College of Cardiology, American Heart Association, and European Society of Cardiology.^14)^ This study was approved by the institutional ethics committee (REC No. CRC-62-040), and informed consent was obtained from all patients. The medical records were retrieved and reviewed for preoperative demographic characteristic, operative details, perioperative outcomes, and follow-up data from the outpatient visits, including the information on cardiac rhythm, medical status, echocardiography results, follow-up interventions, and clinical conditions.

**Fig. 1:**
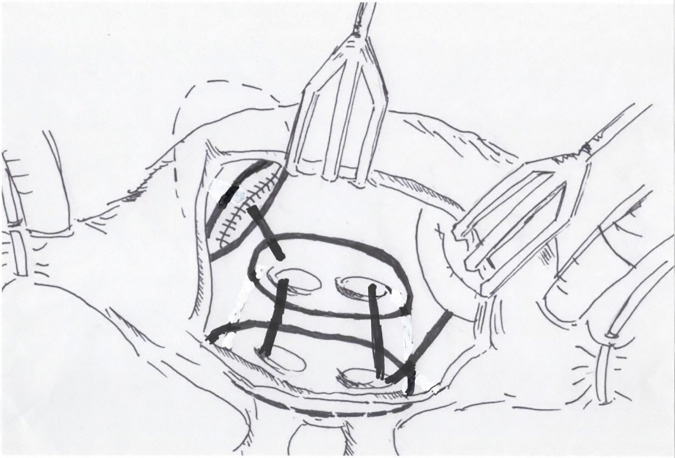
Picture of the left atrial lesion set of RFA consisting of isolation of the left and right pulmonary veins, connecting lesions from the left to right pulmonary orifices, the left superior pulmonary vein to an appendage, around the orifice of the appendage, and from the right inferior pulmonary vein to the mitral anulus. RFA: radiofrequency ablation

**Fig. 2:**
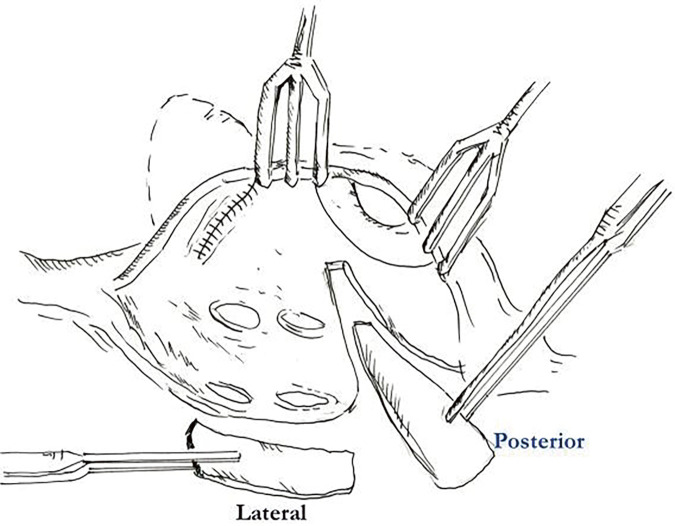
Picture of left atrial reduction technique. The left atrial lateral wall is excised after the interatrial groove is dissected by electrical cautery. The posterior wall is triangularly resected by making an incision from the lower orifice of right inferior pulmonary vein continuing from the previous incision parallel to posterior mitral anulus.

**Fig. 3:**
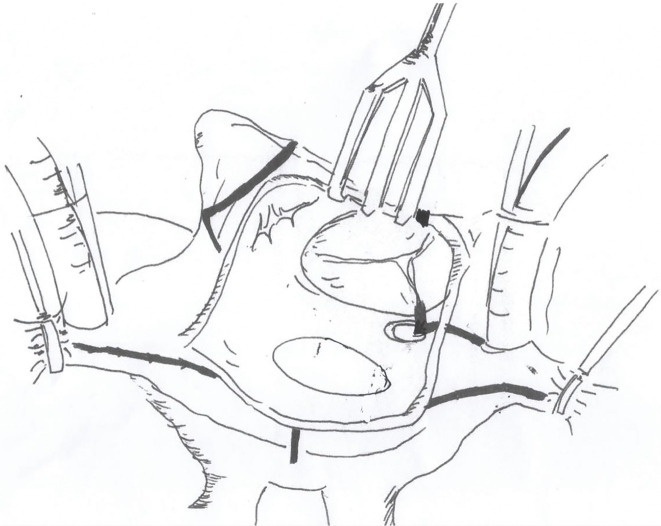
Picture of the right atrial lesion set including superior and inferior vena cava lines, around a right atrial appendage, from the end of transverse incision to the sulcus terminalis, from another end of incision to tricuspid anulus at 12 o’clock, from 9 o’clock of the anulus to the appendage, and from the anulus at poster septal commissure to coronary sinus and continuing to inferior vena cava.

The procedures were performed through median sternotomy incision. An ascending aortic and bicaval cannulations were conducted for cardiopulmonary bypass under moderate hypothermia (28°C–32°C). A cold blood cardioplegia was perfused via aortic root and repeated every 20 min. The mitral valve was approached posterior to an interatrial groove (Sondergaard’s incision). The modified Maze procedure used an irrigated radiofrequency ablation (RFA) device (Cardioblate; Medtronic, Minneapolis, MN, USA) with the electric generator set at 25 W. The ablation was created by either unipolar RFA or combined bipolar RFA with carbon dioxide cryoablation (140 Cryo Unit; Spembly Medical, Andover, UK). The left side ablation lines consisted of ablation of both superior and inferior pulmonary veins, connecting lines between the left and right pulmonary veins as a box lesion, around the orifice of the LA appendage, the left superior pulmonary vein to the appendage, and the right inferior pulmonary vein to the mitral annulus (**Fig. 1**). An importance was every ablation line must have crisscrossed each other to make sure the complete ablation line. The left atrial appendage was amputated or double-layer sutured from the endocardial surface using 4/0 polypropylene at its orifice. In 110 (27.19%) cases, the LA size was reduced by triangular resection of posterior wall just inferior to the right inferior pulmonary vein orifice parallelling the mitral annulus and pointing to the lower rim of the left inferior pulmonary vein, as described by Romano and associates,^15)^ which was combined with lateral excision adjacent to the interatrial groove in 262 (64.5%) cases (**Fig. 2**). For the right side, the ablation lines were carried out to both venae cavae, from the posteroseptal commissural annulus to the lower rim of the coronary sinus orifice and continuing to the inferior vena cava, tricuspid annulus at 9 o’clock to the right atrial appendage, at 12 o’clock to right atriotomy incision, and circumferential around the right atrial appendage (**Fig. 3**). In the case of bipolar RFA, carbon dioxide cryoablation at −70°C for 2 min was applied between the coronary sinus and the mitral annulus for the left side and at the tricuspid isthmus line between the coronary sinus orifice and the tricuspid annulus and inferior vena cava. The lesion patterns of both monopolar and bipolar RFA were the same.

Heart rhythm was monitored continuously for 48 h postoperatively, and a 12-lead EKG was performed after the operation. Follow-up EKGs were also done on days 1 and 7, and at the time of follow-up visits at 1, 3, 6, 9, and 12 months, and every 6 months thereafter. Furthermore, 24-h Holter monitoring was performed at 6 months according to the postoperative protocol. Recurrent AF was defined as any episode of AF, atrial flutter, or atrial tachycardia lasting more than 30 s. Early failure was identified if arrhythmias occurred within the first 6 months postoperatively, while late failure referred to arrhythmias detected thereafter. Amiodarone (100 mg/day) was prescribed for 6 months after the operation except in cases of bradycardia (<60 beats per min) was present. An anticoagulant with warfarin was continued for 6 months and then changed to aspirin (80 mg/day) as lifelong therapy if no AF was detected by Holter monitor. For AF recurrence after 1 year, unresponsive to medical treatment, electrical cardioversion was performed following a transthoracic echocardiogram confirming the absence of cardiac thrombi.

## Statistical analysis

Continuous variables were shown as mean and standard deviation. The Chi-square test and independent *t*-test were utilized for categorical and continuous variables, respectively. Survival and freedom from events were described with Kaplan–Meier curves and compared with Log-rank test. Bivariate and multivariable analyses were performed to explore predicting factors of postoperative AF and overall survival (OS) using the Cox proportional hazard model. Proportional hazards assumptions were approved by testing the scaled Schoenfeld residuals with the global PH-Test. Factors with a p-value of <0.05 in bivariate analyses were considered for multivariable modelling, which was performed by stepwise backward selection (p-value threshold <0.05) to calculate the adjusted hazard ratio for postoperative AF and OS. All statistical tests were carried out using Stata IC15 (Stata Corp, 2017, College Station, TX, USA).

## Results

A retrospective study for AF surgery of 1369 patients at the Central Chest Institute of Thailand from June 2004 to December 2022 was conducted with variety of technics from pulmonary vein isolation, left side modified RFA Maze, and biatrial modified RFA Maze. Of these, 29.7% (406 patients) underwent mitral valve surgery concomitant with modified biatrial Maze operation by a single surgeon during a 17-year period with a median follow-up time of 100 months (inter-quartile range: 51,149 months), with a 97% (394/406 patients) complete follow-up patients’ rate. The mean patients’ age was 53 years and females slightly predominated at approximately 57%. Rheumatic mitral valve pathology was the most common etiology (59%), followed by degenerative (40%) and congenital or infective endocarditis (<1%). Preoperatively, 80% of patients were on digoxin and warfarin. All surviving patients underwent EKG and 24-h Holter monitoring at 6 months, with 12-lead EKG at subsequent follow-ups. Mitral valve repair was performed in approximately 57% of cases, while mechanical and bioprosthetic valve replacements accounted for 37.6% and 4.9%, respectively.

Biatrial modified Maze was carried out in all patients using monopolar RFA in 59.6% and a combination of bipolar and cryoablation or bipolar and monopolar RFA in 40.3%. LA reduction was performed in 91.6% (372 patients) of patients, reducing the mean LAD from 54.4 mm pre-operation to 42.4 mm post-operation (**Tables 1** and **2**).

**Table 1:** Baseline characteristics

	Total	Freedom from AF*	AF**	p-value
	N = 406	N = 297	N = 109	
Mean age, years, (SD)	53.22 (±11.32)	53.33 (±11.55)	52.91 (±10.73)	0.74
Gender, male (%)	174 (42.86%)	130 (43.77%)	44 (40.37%)	0.54
**Etiology**				0.065
Degenerative, n (%)	164 (40.39%)	130 (43.77%)	34 (31.19%)	
Rheumatic, n (%)	238 (58.62%)	163 (54.88%)	75 (68.81%)	
IE, n (%)	1 (0.25%)	1 (0.34%)	0 (0.00%)	
Congenital, n (%)	3 (0.74%)	3 (1.01%)	0 (0.00%)	
**NYHA **				0.99
Class I, n (%)	6 (1.48%)	4 (1.35%)	2 (1.83%)	
Class II, n (%)	231 (56.90%)	169 (56.90%)	62 (56.88%)	
Class III, n (%)	161 (39.66%)	118 (39.73%)	43 (39.45%)	
Class III plus, n (%)***	8 (1.97%)	6 (2.02%)	2 (1.83%)	
History of stroke, n (%)	53 (13.05%)	36 (12.12%)	17 (15.60%)	0.36
Mean duration AF months	24 (10–36)	23 (9–36)	30 (14–41)	0.005
Mean EF (%), (SD)	59.62 (±9.98)	59.97 (±10.34)	58.67 (±8.89)	0.25
Mean LA size mm, (SD)	54.43 (±7.40)	53.80 (±7.30)	56.14 (±7.45)	0.005
LA clot, n (%)	65 (16.01%)	43 (14.48%)	22 (20.18%)	0.16
LAP mmHg, (SD)	17.03 (±6.73)	17.12 (±6.55)	16.76 (±7.24)	0.65
PAP mmHg, (SD)	28.76 (±8.83)	29.06 (±8.96)	27.90 (±8.42)	0.32

* Freedom from AF: success group, ** AF: failed group, ***Class III plus: less than class IV

AF: atrial fibrillation; IE: infective endocarditis; NYHA: New York Heart Association; EF: ejection fraction; LA: left atrium; LAP: mean left atrial pressure; PAP: systolic pulmonary artery pressure

**Table 2:** Procedure and outcomes

	Total	Freedom from AF*	AF**	p-value
	N = 406	N = 297	N = 109	
**Operation**				0.017
MV repair, n (%)	233 (57.39%)	181 (60.94%)	52 (47.71%)	
MV replacement, n (%)	173 (42.61%)	116 (39.06%)	57 (52.29%)	
Mechanical V, n (%)	153 (37.6%)	100 (33.6%)	53 (48.6%)	
Bioprosthetic V, n (%)	20 (4.9%)	16 (5.3%)	4 (3.6%)	
**Concomitant procedure**				0.65
CABG, n (%)	20 (12.82%)	13 (11.50%)	7 (16.28%)	
Tricuspid valve surgery, n (%)	125 (80.13%)	93 (82.30%)	32 (74.42%)	
ASD closure, n (%)	4 (2.56%)	3 (2.65%)	1 (2.33%)	
AVR, n (%)	7 (4.49%)	4 (3.54%)	3 (6.98%)	
LA reduction, n (%)	372 (91.63%)	273 (91.92%)	99 (90.83%)	0.72
**Energy source**				0.99
Monopolar RF, n (%)	242 (59.61%)	177 (59.60%)	65 (59.63%)	
Bipolar plus monopolar or	164 (40.39%)	120 (40.40%)	44 (40.37%)	
Cryoablation, n (%)				
**Outcomes**				
Hospital mortality, n (%)	5 (1.23%)	3 (1%)	2 (1.8%)	
30-day mortality, n (%)	13 (3.2%)	7 (2.4%)	6 (5.5%)	
Post op EF (%), (SD)	59.42 (±10.90)	60.07 (±11.20)	57.74 (±9.92)	0.070
Postop LA size mm (SD)	42.40 (±6.78)	41.18 (±6.44)	45.50 (±6.66)	<0.001
Early failure, n (%)			26 (23.85%)	
Late failure, n (%)			83 (76.15%)	
PPM implantation, n (%)	11 (2.72%)	8 (2.70%)	3 (2.75%)	0.98
Neuro complication at F/U				0.01
Hemorrhage, n (%)	26 (6.4%)	14 (4.7%)	12 (11%)	
Infarction, n (%)	40 (9.9%)	14 (4.7%)	26 (23.8%)	

*Freedom from AF: success group, **AF: failed group

AF: atrial fibrillation; MV: mitral valve; CABG: coronary artery bypass graft; ASD: atrial septal defect; AVR: aortic valve replacement; LA: left atrium; RF: radiofrequency ablation; EF: ejection fraction; PPM: permanent pacemaker; F/U: follow-up

**Fig. 4:**
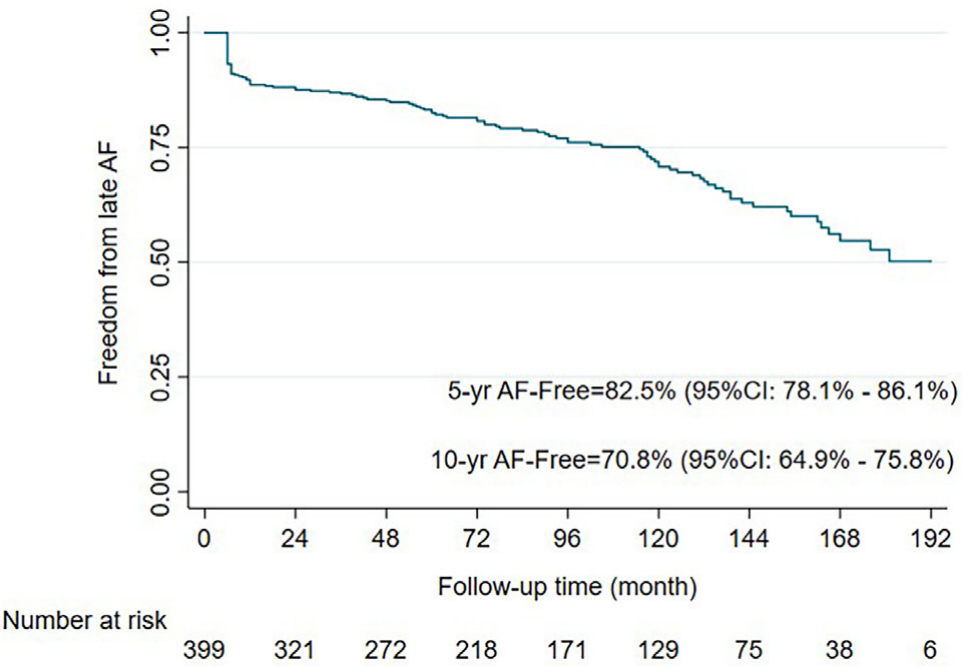
Kaplan–Meier curve of freedom from AF during 16 years of follow-up. AF: atrial fibrillation

**Fig. 5:**
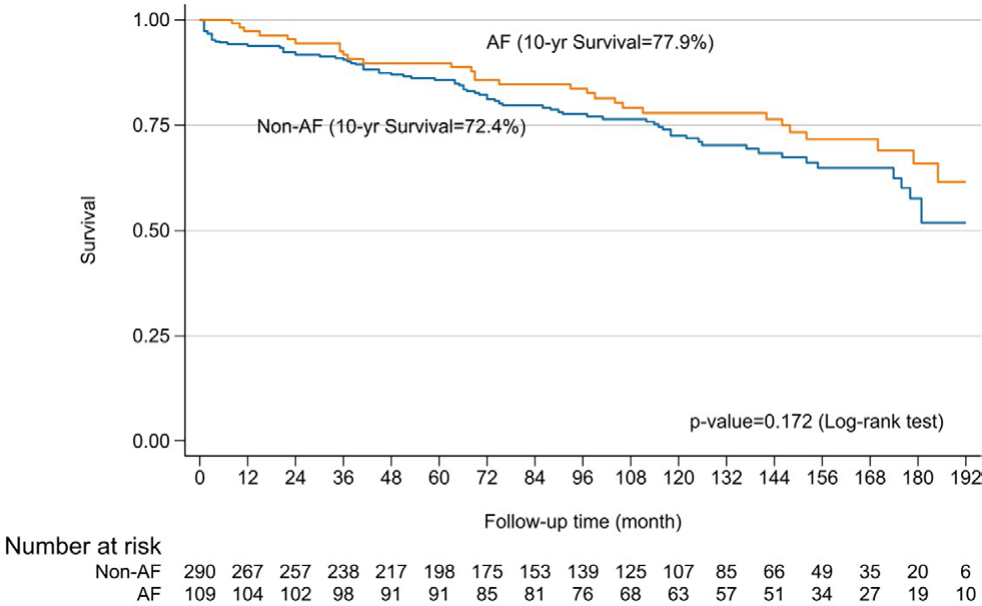
Kaplan–Meier curves of survival at 16 years for patients with AF and non-atrial fibrillation. AF: atrial fibrillation

**Fig. 6:**
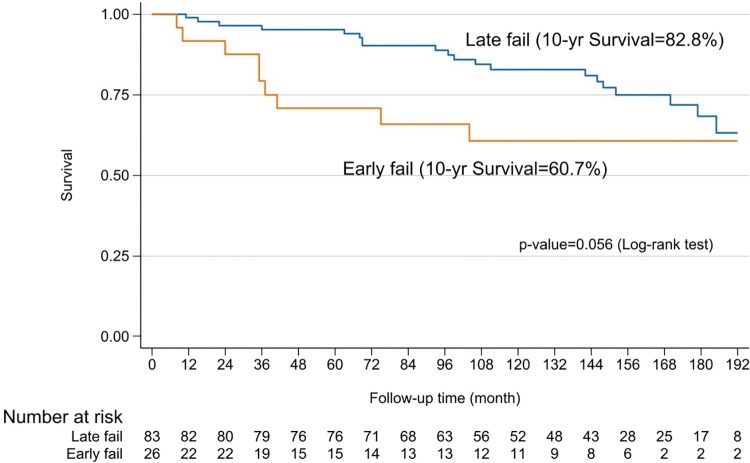
Kaplan–Meier curves of survival comparing early and late failed after returning to SR from the procedure. SR: sinus rhythm

Hospital mortality occurred in 5 cases (1.2%) due to a low cardiac output, with two cases being related to the ablation procedures by right coronary injury proved by coronary angiography and autopsy. Another 8 cases of 30-day mortality occurred after hospital discharge due to ventricular arrhythmia (2 cases), cardiac tamponade (2 cases), cerebral hemorrhage (1 case), warfarin overdose (2 cases), and unknown (1 case). Sixteen deaths occurred within 1 year (4 due to cardiac causes and 5 due to cerebral complication), and 83 late deaths occurred predominantly from cardiac (15 cases), cerebral causes (24 cases), or warfarin overdose (17 cases) complications. Overall freedom from AF at 5, 10, and 15 years was 82.5%, 70.8%, and 52.7%, respectively (**Fig. 4**). However, the survival of patients who continued NSR compared to patients who had relapse AF were not significantly different at 5, 10, and 15 years (90% vs. 78%, 77.9% vs. 72.4%, and 64% vs. 57%; p = 0.172) (**Fig. 5**). Nonetheless, those who failed to maintain NSR had a higher chance of having neurological complications, including cerebral hemorrhage and infarction than patients who had NSR (11% vs. 4.7% and 23.8% vs. 4.7%; p = 0.01) (**Table 2**). A long duration of preoperative AF demonstrated was a significant factor in the AF group (30 vs. 23 months; p = 0.005). Patients who remained in AF after undergoing the modified Maze procedure had significantly larger LAD both preoperatively and postoperatively compared to those in non-AF group (56.1 mm vs. 53.8 mm; p = 0.005; and 45.5 mm vs. 41.1 mm; p <0.001) (**Tables 1** and **2**). Only 2% of patients who had converted from AF continued to receive an antiarrhythmic drug. Among Maze procedure failures, 26 patients (23.5%) experienced early failure, while 83 patients (76.5%) were late failures (**Table 2**), with most AF relapses occurring after 10 years (**Fig. 4**). While there was a trend toward lower survival in the early failure group, this was not statistically significant (70.8% vs. 95.2%; p = 0.056) (**Fig. 6**). Approximately 85% of the patients with NSR conversion were free of antiarrhythmic drugs. During the follow-up, 11 patients (2.7%) required permanent pacemaker implantation.

**Fig. 7:**
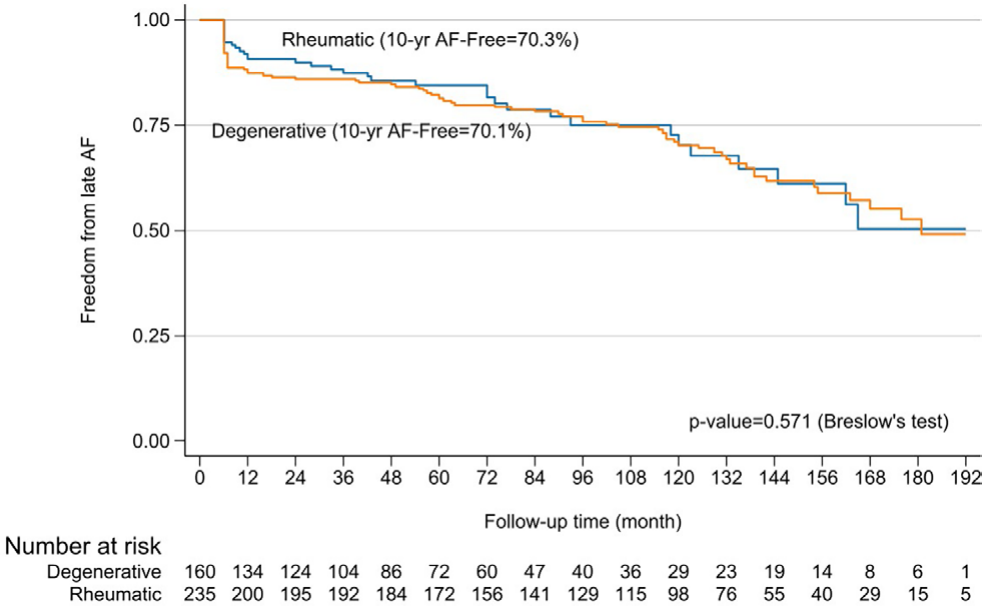
Kaplan–Meier curves of freedom from AF comparing between rheumatic and degenerative mitral valve problems. AF: atrial fibrillations

**Fig. 8:**
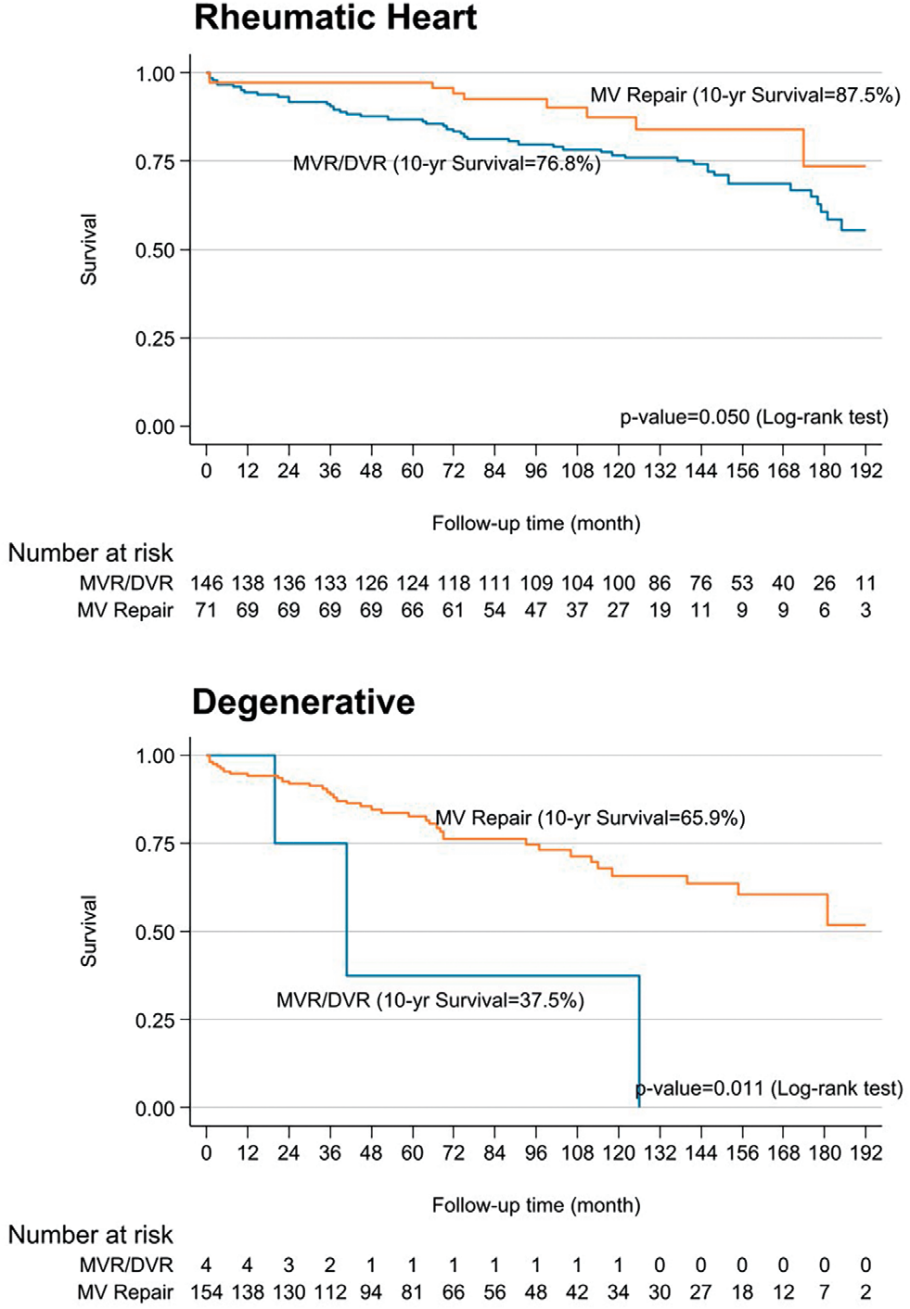
Kaplan–Meier curves demonstrate survival of patients in both major origin causes MV Repair is significant survival than MVR (DVR). MV Repair: mitral valve repair; MVR: mitral valve replacement

Patients with rheumatic mitral valve disease were younger and included fewer males compared to those with degenerative pathology (49.8 vs. 58.2 years; 26% vs. 67%; p <0.001 for both). Stenotic lesions were more prevalent in the rheumatic group (77%), and they had a higher prevalence of prior stroke (18.4% vs. 5.4%; p <0.001). Rheumatic patients also had lower ejection fractions (57.6% vs. 62.6%; p <0.001) and higher LA and systolic pulmonary artery pressures (19 vs. 14 mmHg; p <0.001; and 29.7 vs. 27.5 mmHg; p = 0.03). Mitral valve repair was more successful in degenerative pathology (96%) compared to rheumatic cases (29%) (p <0.001) (**Table 3**). No significant differences in perioperative outcomes or freedom from AF were observed between rheumatic and degenerative groups at 10 and 15 years (70.3% vs. 70.1% and 53.4% vs. 52%, respectively) (**Fig. 7**). However, mitral valve repair showed a trend toward higher 10-year survival compared to valve replacement in both groups (87.5% vs. 76.8%; p = 0.05 and 65.9% vs. 37.5%; p = 0.01) (**Fig. 8**).

**Table 3:** Degenerative vs. rheumatic

	Degenerative	Rheumatic	p-value
	N = 164	N = 238	
Mean age, years, (SD)	58.27 (±10.49)	49.87 (±10.59)	<0.001
Gender, male, n (%)	110 (67.07%)	62 (26.05%)	<0.001
**NYHA**			0.16
Class I, n (%)	5 (3.05%)	1 (0.42%)	
Class II, n (%)	94 (57.32%)	135 (56.72%)	
Class III, n (%)	61 (37.20%)	98 (41.18%)	
Class III plus, n (%)*	4 (2.44%)	4 (1.68%)	
Hx stroke, n (%)	9 (5.49%)	44 (18.49%)	<0.001
Duration AF months, (SD)	24 (12–36)	24 (10–36)	0.50
EF, (%)	62.67 (±10.17)	57.62 (±9.33)	<0.001
LA size, mm, (SD)	54.55 (±7.78)	54.55 (±7.03)	1.00
LA clot, n (%)	1 (0.61%)	64 (26.89%)	<0.001
LAP mmHg, (SD)	149.02 (±5.69)	19.00 (±6.67)	<0.001
PAP mmHg, (SD)	27.52 (±8.75)	29.71 (±8.84)	0.032
**Operation**			<0.001
MV repair, n (%)	158 (96.34%)	71 (29.83%)	
MV replacement, n (%)	6 (3.66%)	167 (70.17%)	
Mechanical V, n (%)	4 (66.67%)	149 (89.22%)	
Bioprosthetic V, n (%)	2 (33.33%)	18 (10.78%)	
**Concomitant procedure**			0.22
CABG, n (%)	11 (13.10%)	9 (13.04%)	
Tricuspid valve surgery, n (%)	71 (84.52%)	53 (76.81%)	
ASD closure, n (%)	1 (1.19%)	2 (2.90%)	
AVR, n (%)	1 (1.19%)	5 (7.25%)	
LA reduction, n (%)	150 (91.46%)	219 (92.02%)	0.84
**Energy source**			
Monopolar RF, n (%)	98 (59.76%)	142 (59.66%)	0.99
Bipolar plus monopolar or cryoablate, n (%)	66 (40.24%)	96 (40.34%)	
**Outcomes**			
Hospital mortality, n (%)	2 (1.22%)	3 (1.26%)	0.97
30-day mortality, n (%)	6 (3.66%)	6 (2.5%)	0.36
Post op EF n (%), (SD)	59.79 (±11.10)	59.24 (±10.85)	0.65
Post op LA size mm, (SD)	41.23 (±6.84)	43.27 (±6.61)	0.005
Post op AF, n (%)	34 (20.73%)	75 (31.51%)	0.571
Early failure, n (%)	8 (23.53%)	18 (24%)	0.96
Late failure, n (%)	26 (76.47%)	57 (76%)	
Permanent pacemaker, n (%)	4 (2.44%)	7 (2.95%)	0.76

*Class III plus: less than Class IV

NYHA: New York Heart Association; Hx: History; AF: atrial fibrillation; EF: ejection fraction; LA: left atrium; LAP: mean left atrial pressure; PAP: systolic pulmonary artery pressure; MV: mitral valve; CABG: coronary artery bypass graft; ASD: atrial septal defect; AVR: aortic valve replacement; LA: left atrium; RF: radiofrequency ablation

Preoperative and perioperative parameters were analyzed for their impact on postoperative AF and survival. Significant predictors of postoperative AF included longer preoperative AF duration (p <0.001), LAD >50 mm pre- and postoperatively (p = 0.003, p = 0.045), associated tricuspid valve disease (p = 0.049), and AF on postoperative day 7 (p <0.001). Age, rheumatic etiology, postoperative LVEF <40%, concomitant tricuspid valve surgery, and postoperative day-7 AF were univariable predictors of survival. However, multivariable analysis identified only age and postoperative ejection fraction <40% as independent survival predictors (p <0.001) (**Tables 4** and **5**).

**Table 4:** Predictor of freedom from AF

Factors	Univariate analysis				Multivariate analysis			
	Crude HR	95% CI		p-value	Adjusted HR	95% CI		p-value
		Lower	Upper			Lower	Upper	
Age (years) >60	1.01	0.64	1.61	0.958				
Female	1.01	0.69	1.48	0.96				
Rheumatic vs. degenerative	1.06	0.7	1.6	0.779				
Duration	1.01	1.01	1.02	<0.001	1.011	1.006	1.017	<0.001
Pre operative EF (%) >40	3.91	3.91	3.91	3.91				
Post operative EF (%) >40	1.77	0.72	4.38	2.216				
**Operation**								
MV repair	1.06	0.38	2.94	0.908				
MVR (mechanical)	1.24	0.45	3.48	0.677				
DVR	1.03	0.3	3.53	0.963				
Bioprosthesis	1.0							
Pre op LAD (mm) >60	1.75	1.15	2.66	0.009				
Pre op LAD (mm) >50	2.21	1.38	3.53	0.001	1.73	1.05	2.86	0.031
Post op LAD (mm) >50	2.53	1.6	4.01	<0.001	1.65	1.01	2.69	0.045
LAP (mmHg) <15	1.31	0.87	1.96	0.198				
PAP (mmHg) <40	1.4	0.67	2.92	0.367				
**LA reduction**								
No reduction	1.26	0.65	2.45	0.496				
Posterior	1.21	0.78	1.88	0.394				
Posterior and lateral	1							
**Technique of ablation**								
Monopolar	1.85	0.57	5.95	0.303				
Bipolar with cryoablation	2.29	0.7	7.47	0.17				
Combine mono and bipolar	1							
Associated tricuspid valve	1.53	1	2.35	0.049	1.56	1	2.42	0.049
AF on day 7	3.16	2.16	4.64	<0.001	2.73	1.81	4.12	<0.001

HR: hazard ratio; EF: ejection fraction; MVR: mitral valve replacement; DVR: double valve replacement; LAD: left atrial diameter; LAP: mean left atrial pressure; PAP: systolic pulmonary artery pressure; AF: atrial fibrillation

**Table 5:** Predictor of survival

Factors	Univariate analysis				Multivariate analysis			
	Crude HR	95% CI		p-value	Adjusted HR	95% CI		p-value
		Lower	Upper			Lower	Upper	
Age (years) >60	2.71	1.81	4.05	<0.001	2.65	1.69	4.15	<0.001
Female	0.97	0.66	1.44	0.895				
Rheumatic vs. degenerative	1.62	1.09	2.41	0.017	1.3	0.82	2.06	0.268
Pre operative EF (%) >40	1.14	0.36	3.59	0.823				
Post operative EF (%) >40	4.01	2.4	11.6	<0.001	3.76	1.8	7.75	<0.001
**Operation**								
MV repair	1	0.45	2.22	0.997				
MVR (mechanical)	1.05	0.47	2.33	0.911				
DVR	1							
Bioprosthesis	1.66	0.58	4.74	0.347				
Pre op LAD (mm) >60	0.81	0.49	1.35	0.423				
Post op LAD (mm) >50	1.11	0.61	2.01	0.729				
LAP (mmHg) <15	1.49	1	2.23	0.051	1.08	0.69	1.67	0.735
PAP (mmHg) <40	1.28	0.61	2.65	0.514				
**LA reduction**								
No reduction	1.38	0.76	2.49	0.286	1.08	0.5	2.36	0.842
Posterior	0.57	0.33	0.98	0.042	0.6	0.31	1.14	0.119
Posterior and lateral								
**Technique of ablation**								
Monopolar	1.44	0.45	4.66	0.542				
Bipolar with cryoablation	1.8	0.55	5.88	0.332				
Associated tricuspid valve	1.44	0.93	2.22	0.101				
AF on day 7	1.58	1.06	2.37	0.026	1.53	0.99	2.37	0.058

HR: hazard ratio; EF: ejection fraction; MVR: mitral valve replacement; DVR: double valve replacement; LAD: left atrial diameter; LAP: mean left atrial pressure; PAP: systolic pulmonary artery pressure; AF: atrial fibrillation

**Fig. 9:**
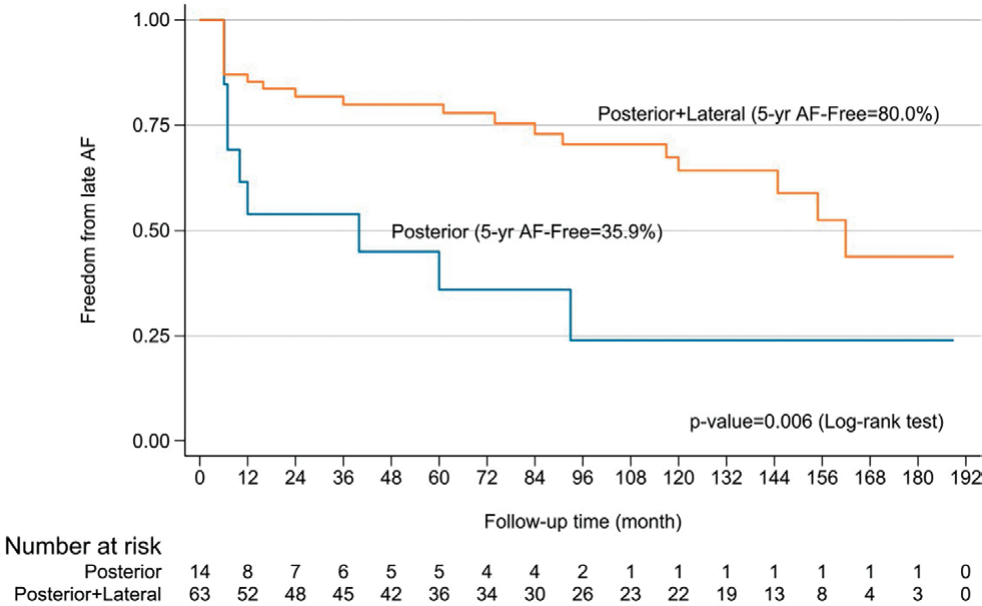
Kaplan–Meier curves show freedom from AF for patients who had technic of posterior reduction and who had posterolateral resection for left atrial size >60 mm. AF: atrial fibrillation

Although the outcomes were not different between the resections of the left atrium, when the LAD >60 mm in diameter all left atriums reduced in size either for the posterior (Romano et al.^15)^) or our modified posterolateral resection (**Fig. 2**). The authors compared posterior and aggressive posterolateral resections in patients with a left atrial diameter >6 cm. Posterior resection reduced the mean size from 65.1 to 51.5 mm, while posterolateral resection achieved a reduction from 63.4 to 45.5 mm. The freedom from AF in this group showed a statistically significant difference (p = 0.006) (**Fig. 9**).

## Discussion

AF, as a consequence of mitral valve pathologies, was found in 40%–60% of cases,^3)^ leading to worsened outcomes such as palpitations, an increased risk of thromboembolic events, and atrial remodeling, which can contribute to mitral and tricuspid regurgitation.^2)^ The classical Cox-Maze procedure, introduced by Dr. James Cox in 1987, remains the standard treatment for AF. The procedure evolved into the Cox-Maze III, which used the cut-and-sew technique to isolate electrical segments, and further refined into the modified Cox-Maze (Cox-Maze IV) by Henn and colleagues in 2015.^16)^ The modified technique is now approved as the standard approach in the Society of Thoracic Surgeons guidelines 2017.^5)^ Its effectiveness in restoring NSR varies, with reported success rates of 63%–77% in both valvular and non-valvular AF.^17)^ However, most studies reported outcomes in less than 10 years’ outcome.

The present study explores the long-term outcomes of combining mitral valve surgery with the modified RFA Maze procedure in 406 patients. Freedom from AF was observed at 5, 10, and 15 years in 82.5%, 70.8%, and 52.7% of patients, respectively. These results are consistent with other studies showing a tendency for AF recurrence over time following successful conversion to NSR. For instance, freedom from AF at 10 years was 63%, with 51% at 20 years.^13)^ In contrast, the standard cut-and-sew Cox-Maze procedure showed a freedom from AF of 68% at 5 years, which declined to 41% at 10 years.^6)^ Importantly, the main goal of the Cox-Maze procedure is to improve survival. A previous report found that patients who maintained NSR post-operation had better survival rates at 10 years compared to those with recurrent atrial arrhythmias (84% vs. 77%; p = 0.03).^9)^ In this study, the 10- and 15-year survival rates for patients who achieved NSR were 77.9% and 64%, respectively, compared to 72.4% and 57% for those with AF recurrence (p = 0.172). Despite no significant survival difference, neurological outcomes were better for patients who remained in NSR (11% vs. 4.7% and 23.8% vs. 4.7%, respectively; p = 0.01). These data were consistent with other reports,^18,19)^ showing no significant survival difference between these two groups but a reduction in adverse neurological outcomes (p <0.0001 and p = 0.044 respectively). This supports the notion that a successful Cox-Maze procedure reduces severe complications, even if survival rates are similar. Regarding the timing of failure, early failure of the Maze procedure was associated with lower 10-year survival rates compared to late failure (60.7% vs. 82.8%), although this difference was not statistically significant (p = 0.056) (**Fig. 6**).

Permanent pacemaker implantation requirement for sinoatrial node dysfunction after the Cox-Maze operation is one of interesting points. According to a recent meta-analysis^20)^ reported permanent pacemaker insertion ranged from 0% and 14.2% in patients undergoing concomitant valve and Maze procedure. In this study, a permanent pacemaker was implanted in 11 (2.7%) patients. One of the major etiologies of combined mitral valve and AF is rheumatic heart disease, and while one paper reported rheumatic disease is a predictor for failure of the Cox-Maze procedure,^7)^ other studies,^8–11)^ including a prospective randomized trial,^12)^ demonstrated no significant different in freedom from AF between patients with rheumatic and nonrheumatic mitral valve disease.

It is generally accepted that mitral valve repair generally leads to better short- and long-term outcomes than valve replacement when good repair is performed in SR circumstance. This study had a repair rate of 96% for degenerative and 29% for rheumatic disease. There was no significant difference in 10-year freedom from AF between patients undergoing repair or replacement (70.1% vs. 70.3% at 10 years, p = 0.571) (**Fig. 7**), consistent with other studies.^18,21)^ However, the repair group exhibited significantly higher overall survival rates at 10 years in both rheumatic (87.5% vs. 76.8%, p = 0.05) and degenerative (65.9% vs. 37.5%, p = 0.011) cases (**Fig. 8**).

Several factors have been identified as predictors of recurrent AF,^12,13,21,22)^ including older age, rheumatic heart disease, peripheral vascular disease, prolonged AF duration, LA size, cardiothoracic ratio, and the absence of NSR at hospital discharge. Studies have shown that longer AF durations contribute to advanced atrial remodeling, which is linked to late AF recurrence.^23)^ Preoperative large LA, particularly in patients with diameters >60 mm, had a high chance of failure.^21,22)^ A systematic review further concluded that a LAD >60 mm is 100% sensitive for predicting Maze failure.^24)^ In addition, one study^10)^ reported this independent predicting number was 50 mm. In our series, the mean preoperative LAD was 54.4 mm, and 77 patients (19%) had LAD >60 mm. To address this issue, several LA reduction techniques have been introduced,^15,25–28)^ as well as one positive outcomes from a randomized study.^29)^ In this study, 91% of the cases underwent LA reduction, which resulting in a mean postoperative LAD of 42.3 mm. Both preoperative and postoperative LAD >50 mm were identified as significant predictors of AF recurrence (p = 0.001 univariate, p = 0.031 multivariate; p <0.001 univariate, p = 0.045 multivariate, respectively). Furthermore, when the LA size is larger than 60 mm, combined posterior and lateral LA resection proved better freedom from AF than posterior excision alone (p = 0.006) (**Fig. 9**). These findings are consistent with previous studies that emphasize the role of LA reduction in improving long-term outcomes. In addition to LA size, our study identified a long history of AF, concomitant tricuspid surgery, and the presence of AF on day 7 as significant prognostic factors for long-term success.

### Limitations of the study

As this was a retrospective study, comparisons between groups may not have been entirely appropriate due to inherent limitations in study design and potential biases. Additionally, most of the patients in this cohort had limited education, which made it challenging for them to recognize and report episodes of AF accurately. Data regarding AF duration were collected indirectly, such as from medical history and warfarin prescription records. Consequently, the actual duration of AF is longer than what was documented by the authors.

## Conclusions

The addition of the modified Maze procedure to mitral valve surgery is a viable surgical option for treating AF associated with mitral valve disease. While the rate of recurrent AF gradually increases over time, particularly beyond a 10-year follow-up period, the outcomes of this combined approach were comparable across different etiologies of valve disease, including rheumatic origins. Mitral valve repair demonstrated better survival outcomes than valve replacement for both rheumatic and degenerative causes. AF recurrence was influenced by factors such as preoperative AF duration, pre- and postoperative LAD >50 mm, associated tricuspid valve surgery, and the presence of AF on postoperative day 7.

For patients with an LAD >60 mm, posterolateral LA reduction yielded significantly better freedom from AF than posterior reduction alone. Predictors of survival included age >60 years and postoperative LVEF <40%. Although survival rates between the AF-free (NSR) and recurrent AF groups were not significantly different, neurological complications were notably less frequent in the NSR group (p = 0.01).

## Acknowledgments

The authors gratefully thank all patients who were included in this study for their cooperation to long regularly follow-ups at Central Chest Institute of Thailand, Ms. Pimrapat Gebert for her statistical analysis, and staff members of department of Cardiology for performing investigations during the study.

## Declarations

### Ethic approval and consent to participate

This study was approved by the Central Chest Institutional of Thailand ethics committee REC No. CRC-62-0404. Informed consent was obtained from the patients for this study.

### Funding

This study did not receive specific grant from any funding agency neither institute nor commercial agency.

### Conflict of interests

The authors declared no conflict of interest.

### Data availability

The data that support the findings of this study are available from the corresponding author upon reasonable request.

### Authors’ contributions

Contributions of this study were comprised as follows: CK was a surgeon who did all cases of Modified Maze procedure concomitant with mitral valve operation, reviewed, and rewrote a revised manuscript. PP and SW assisted in collecting data and analysis. PP and SW were writers of the primary manuscript. PL was an artist who collected patients’ follow-up and existence of life status including cause of death from the National Database of Ministry of Interior Affair of Thailand. All authors have read and approved the final version of the manuscript.
